# Agreement among Four Prevalence Metrics for Urogenital Schistosomiasis in the Eastern Region of Ghana

**DOI:** 10.1155/2016/7627358

**Published:** 2016-12-18

**Authors:** Karen Claire Kosinski, Alexandra V. Kulinkina, David Tybor, Dickson Osabutey, Kwabena M. Bosompem, Elena N. Naumova

**Affiliations:** ^1^Department of Community Health, Tufts University School of Arts and Sciences, 574 Boston Avenue, Medford, MA 02155, USA; ^2^Initiative for the Forecasting and Modeling of Infectious Diseases, Tufts University, Medford, MA 02155, USA; ^3^Department of Civil and Environmental Engineering, Tufts University School of Engineering, Medford, MA 02155, USA; ^4^Department of Public Health and Community Medicine, Tufts University School of Medicine, Boston, MA 02111, USA; ^5^Department of Parasitology, Noguchi Memorial Institute for Medical Research (NMIMR), University of Ghana, P.O. Box LG581, Legon, Ghana; ^6^Friedman School of Nutrition Science & Policy, Tufts University, Boston, MA 02111, USA

## Abstract

Few studies assess agreement among* Schistosoma haematobium* eggs, measured hematuria, and self-reported metrics. We assessed agreement among four metrics at a single time point and analyzed the stability of infection across two time points with a single metric. We used data from the Eastern Region of Ghana and constructed logistic regression models. Girls reporting macrohematuria were 4.1 times more likely to have measured hematuria than girls not reporting macrohematuria (CI_95%_: 2.1–7.9); girls who swim were 3.6 times more likely to have measured hematuria than nonswimmers (CI_95%_: 1.6–7.9). For boys, neither self-reported metric was predictive. Girls with measured hematuria in 2010 were 3.3 times more likely to be positive in 2012 (CI_95%_: 1.01–10.5), but boys showed no association. Boys with measured hematuria in 2008 were 6.0 times more likely to have measured hematuria in 2009 (CI_95%_: 1.5–23.9) and those with eggs in urine in 2008 were 4.8 times more likely to have eggs in urine in 2009 (CI_95%_: 1.2–18.8). For girls, measured hematuria in 2008 predicted a positive test in 2009 (OR = 2.8; CI_95%_: 1.1–6.8), but egg status did not. Agreement between dipstick results and eggs suggests continued dipstick used is appropriate. Self-reported swimming should be further examined. For effective disease monitoring, we recommend annual dipstick testing.

## 1. Introduction

Schistosomiasis is a neglected tropical disease (NTD). Surveillance and regular mass drug administration (MDA) are essential in highly endemic Sub-Saharan African countries, even as other countries begin to move towards elimination of the disease using additional primary prevention measures [1]. Substantial efforts were made to provide preventive chemotherapy (praziquantel) to at least 75% of school-aged children at risk of morbidity (WHA54.19). However, the goal was not achieved by the target year of 2010 and more work needs to be done to ensure that Sustainable Development Goal 3.3, end the NTD epidemic, is met by the year 2030 [2]. Specifically, it is necessary to have surveillance strategies that are cost-effective and accurate and these surveillance strategies should be used to determine where, when, and among which demographic groups to deploy MDA and water, sanitation, and hygiene (WASH) interventions [1, 3, 4].

For control of* Schistosoma haematobium*, the WHO currently recommends parasitological methods (finding eggs in urine), morbidity measurements (measured hematuria), or self-reported macrohematuria to identify “high-risk,” “moderate-risk,” and “low-risk” communities [5]. These three metrics are recorded at the level of an individual child and are used to estimate prevalence for a community [5]. However, agreement among these assessment metrics across communities and demographic groups remains to be determined, now that MDA with praziquantel is a widespread practice in school-aged children. Following MDA, light infections (<50 eggs/10 mL urine) tend to be more common, and these light infections can be difficult to diagnose [6, 7]. In a recent review by Knopp et al. [4], the sensitivity and specificity of a variety of diagnostic tests (questionnaires, reagent strips, egg filtration, polymerase chain reaction (PCR), antibody-based assays, miracidium hatching test, and rapid diagnostic tests for* S. haematobium* infection) were discussed; in the public health literature, sensitivity and specificity are widely used measures of diagnostic test accuracy. Knopp et al. [4] found that questionnaires are not appropriate for diagnosis of light or very light infections; they also concluded that the dipstick test for microhematuria and filtration for* S. haematobium* eggs are appropriate for heavy infections, but only moderately appropriate for light infections.

Identification of endemic communities in resource-poor areas is challenging for a number of reasons. First, schistosomiasis is a focal disease [3, 5, 8–10] and communities that are geographically proximal may not have similar urogenital schistosomiasis (UGS) prevalence levels. Second, screening methods have varying levels of sensitivity and specificity [4, 7, 11, 12] and it is difficult to compare prevalence assessed via different screening tools. Third, the most widely used tools for population-level screening are likely to underestimate UGS prevalence following MDA [4, 6, 7, 13–16]. Finally, healthcare systems incur costs (e.g., labor, supplies, and time) with any screening program, and these costs must be minimized [3, 7, 17, 18].

In practice, identification of endemic communities by national health authorities may rely on expert opinion of healthcare workers, researchers, and officials; knowledge about past endemicity; passive case reporting [5]; nonsystematic observations; large-scale maps; or advocacy on the part of well-connected communities and their leaders. Identification can also be influenced by community accessibility and distance from urban areas. In Ghana, a patient with schistosomiasis symptoms who visits a local health facility is likely to be referred to a district-level healthcare facility that maintains stocks of praziquantel and a lab with diagnostic capability; the case is then reported from that district-level facility, which might be quite distant from where the patient actually lives and where the infection was contracted, providing little to no useful information about UGS transmission sites and endemic communities.

In addition to identifying high-prevalence communities, there is a need to identify communities with consistently low prevalence levels in order to reduce unnecessary treatment of populations that are not at risk [8], minimize community fatigue with MDA campaigns, and reduce the likelihood of drug resistant* S. haematobium*.

It is in the best interest of endemic communities and national health systems, particularly in low-income settings with limited resources, if rapid proxies for UGS can be used that correlate strongly with parasitological and morbidity measurements and with true underlying prevalence. We considered three metrics that are well established and widely used (measured hematuria via dipstick;* S. haematobium* eggs via filtration; self-reported macrohematuria) and the fourth that is not in widespread use (self-reported swimming at water contact sites). Swimming and recreational water contact are often associated with schistosomiasis [3, 19] because these activities often involve long contact times with infectious water bodies and a large percentage of skin exposure [20, 21], but other studies have not found the same relationship [22]. We hypothesized that this fourth metric, self-reported swimming behavior, would correlate with the outcomes of other diagnostics methods such as self-reported macrohematuria, measured hematuria, and parasitological methods [3, 21]. Considering the transmission pathways, agreement between measured hematuria and self-reported macrohematuria/swimming is an important validation of the appropriateness of using the two self-reporting methods in the field. Agreement between the two self-reported metrics could be useful in situations where it is preferable to ask schoolchildren whether or not they swim, rather than asking them to report macrohematuria.

With* S. haematobium* infection, there is high potential for reexposure and reinfection [19, 23]. From a public health perspective, reinfection is of interest for several reasons. First, reinfection suggests that individuals may have repeated behaviors [20] from lack of access to acceptable water infrastructure [22], lack of knowledge about schistosomiasis transmission mechanisms, or risky attitudes and practices such as inability/unwillingness to use existing water infrastructure. To test for temporal stability of infection status at the individual level, it is necessary to have a dataset with matched outcome data at multiple time points, which we used in our analyses.

We analyzed longitudinal datasets collected over four years in five different communities in the eastern region of Ghana to (a) assess agreement among four metrics at an individual level (eggs in urine, measured hematuria, self-reported macrohematuria, and self-reported swimming) at a single time point and (b) analyze the stability (i.e., reproducibility and predictability) of infection across two time points when using a single metric (measured hematuria or eggs via filtration). To achieve both objectives, we used logistic regression to assess the relationship between the outcome of interest and the primary predictor, controlling for age, sex, and town of residence. Our longitudinal data, gathered by tracking schoolchildren across two time points, allowed us to assess stability at the individual level, which is indicative of high potential for reinfection.

## 2. Materials and Methods

### 2.1. Study Design

**Figure 1:**
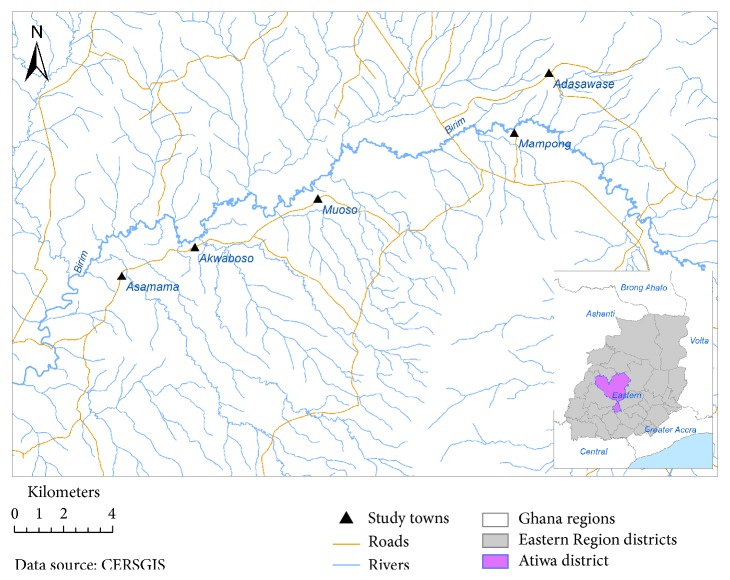
Relative locations of five study communities (black triangles), roads, and rivers; all study communities are located in Atiwa district in the eastern region of Ghana.

The study utilized amalgamation of deidentified secondary data collected from previous projects [24]. The data included UGS prevalence data collected at four different time points and involved schoolchildren in five communities in the eastern region of Ghana (Figure 1). Four different metrics were used (*S. haematobium* eggs via filtration, measured hematuria, self-reported macrohematuria, and self-reported swimming) to collect data through the school system in different years (2008, 2009, 2010, and 2012).

There was no previous MDA with praziquantel in the study communities before 2008. Praziquantel was distributed after screening for infection in each of the studies from which data was drawn. Treatment distribution methods depended on the prevalence of infection and guidance offered by GHS (Tables S1 and S2; see Supplementary Material available online at http://dx.doi.org/10.1155/2016/7627358). Treatment was also administered by schoolteachers with supervision of GHS nurses at some of the schools in Adasawase, Asamama, Mampong, and Bomaa in the summer of 2011 as part of the national deworming exercise.

### 2.2. Study Population and Recruitment

Data were originally collected during several studies that took place between 2008 and 2012 in five communities (Adasawase, Asamama, Akwaboso, Mampong, and Muoso) located in Atiwa district (Figure 1). In these original studies, screening and treatment took place through schools during the summer (May through July) of each study year (Table S1, Supplemental Material). For the present study, we extracted deidentified data from each of these original studies for the analyses conducted here. The process for obtaining permission, conducting community outreach, and recruiting participants was identical for all study communities and is described elsewhere [24]; the Institutional Review Board at Tufts University, Medford, Massachusetts, approved each of the original studies from which the deidentified data were drawn. Between 47.2% and 97.4% of children who were enrolled in school participated in the various studies, depending on the town and year of the study (Table S2, Supplemental Material).

### 2.3. Parasitological and Morbidity Variables

The parasitological metric used here was identification of eggs in urine. The morbidity measurement was the presence of measured hematuria (either micro- or macro-) via dipstick. All urine samples were tested for measureable hematuria via a semiquantitative dipstick test, regardless of whether they were visibly bloody, and categorized as a binary variable with any blood presence, including “trace,” coded as a positive reading [12]. Urine samples were also tested for* S. haematobium* egg presence using filtration methods [24] and categorized as a binary variable with presence of >0.5 eggs/10 mL of urine coded as “1.”

### 2.4. Self-Reported Variables

Self-reported macrohematuria and self-reported swimming were collected via one-on-one verbal interviews in a private location by a native Twi-speaker. Individuals were asked if they have seen macrohematuria within the past week and responses were recorded as “yes” or “no”; for teenage girls, the interviewer clarified that the question was not asking about menstrual blood. In order to assess self-reported swimming, participants were asked, “How often do you normally/currently go to the river?” and “What activities do you do at the river?” Participants were given a list of possible activities from which to choose or mentioned additional activities. The following is an exhaustive list of all activities performed (either from the provided list or mentioned by a participant): swim, play, do laundry, bathe, wash dishes, and fetch water to bring home. Each participant was told that “swimming” meant (a) traditional swimming in which the body is immersed, (b) splashing in the river for recreational purposes, or (c) bathing and occasionally splashing for recreational purposes; Kvalsvig and Schutte [20] similarly found it challenging to distinguish between swimming and washing behaviors. Participants who reported any of these swimming-related activities were recorded as responding “yes” to the question about swimming. During pilot testing of the study questions, we found that, for sociocultural reasons, this series of two questions was more likely to produce an affirmative response about self-reported swimming than simply asking “Do you swim?”.

### 2.5. Longitudinal Assessment

For the longitudinal assessments across two time points, among the 324 children who provided a urine sample in 2010, 243 also provided a sample in 2012 (75% retention). Among the 458 children who provided a urine sample in 2008, 306 also provided a sample in 2009 (67% retention). There were no substantial differences in the sex, age, or town distribution in the samples from which data was drawn for longitudinal analyses (individuals who contributed two data points) as compared to the total population (individuals who contributed one data point and were therefore excluded).

### 2.6. Data Analysis

**Figure 2:**
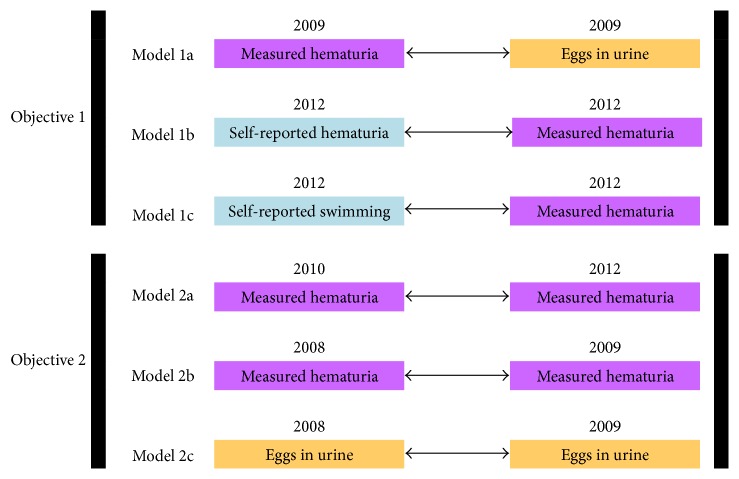
Overall study design: the two study objectives are shown along with the 6 analyses that were performed and the datasets that were used; “eggs in urine” refer to identifying* S. haematobium* eggs in urine samples; “measured hematuria” refers to micro- or macrohematuria via a semiquantitative dipstick; “self-reported macrohematuria” and “self-reported swimming” refer to self-reported presence of macrohematuria and swimming behavior, respectively, by the individual study participant in a private setting and not through hand-raising in a classroom. For logistic regression models, the left column refers to a predictor of interest (*X*) and right column refers to an outcome variable (*Y*).

The study had two main objectives (Figure 2): in the first, we assessed the agreement between different metrics of infection at one time point (models 1a, 1b, and 1c) and in the second, we assessed the temporal stability of infection/reinfection over time as determined by two common metrics: eggs and blood in urine (models 2a, 2b, and 2c). A binary variable was defined for each of these four metrics: presence or absence of eggs in urine, presence or absence of measured hematuria, self-reported macrohematuria, and self-reported swimming (see above). Kendall's Tau, chi-square, and logistic regression models were run separately for boys and girls [25, 26]; logistic regression models utilized age category and town as covariates [19, 21]. Logistic regression models were selected given the ability to model a binary outcome variable and to control for factors such as age and town; the models were used to assess correlation without necessarily implying causation. Descriptive statistics of all variables are shown in Table 1.

We categorized the age variable into three groups: 6–10 years, 11–14 years, and 15+ years, and used the youngest group in the regression models as the reference category. The town variable was also categorized into three groups based on the cross-sectional prevalence of UGS at the first screening conducted in the community. Akwaboso, Mampong, and Muoso were in the first group, representing low UGS prevalence (<10%); Adasawase was in the second group (prevalence close to 20%); and Asamama was in the third group (prevalence close to 50%) (Table S2, Supplemental Material). Additionally, Adasawase was placed in group 2 by itself because a water recreation area (WRA) was constructed there and praziquantel was distributed regularly between 2008 and 2012 [27]. Thus, the inclusion of the location category in the model allowed us to control for town-level UGS prevalence.

Chi-square and Kendall's Tau tests were used to conduct exploratory data analysis; data were stratified by sex, age group, and town (Tables S3 and S4, Supplemental Material). For the exploratory analyses, we conducted post hoc power calculations, restricted to samples collected from boys since this group consistently contained smaller sample sizes than those that involved girls. For the statistical tests conducted on 308 samples, we have sufficient power (>80%) to detect the difference of 6.7% and higher, given that true prevalence is ~25%. Similarly, for the analysis conducted on 292 samples, we have sufficient power to detect the difference of 7%. In measuring an agreement over time, the sample size of 119 and 130 allowed us to detect the difference of 10.5% and 10.1%, respectively. Following the exploratory analyses, we constructed six logistic regression models (R version 3.1.2) (Figure 2) that were formulated as follows:

(1) $$\begin{array}{c} logit\left(\;Y_{i}\;\right)=\beta_{0}+\beta_{1}X_{i}+\beta_{2}{\text{Age}}_{i}+\beta_{3}{\text{Town group}}_{i}, \end{array}$$

where *Y*_*i*_ is an outcome and *X*_*i*_ is a predictor of interest for *i*-child (see details below). All models were also run with the outcome (*Y*_*i*_) and predictor of interest (*X*_*i*_) reversed to test for consistency of association.

#### 2.6.1. Objective 1: Agreement among Diagnostic Metrics

In model 1a, we assessed the agreement between eggs in urine (*Y*_*i*_) and measured hematuria (*X*_*i*_) for data collected in Adasawase and Asamama in 2009. Adasawase had experienced MDA with praziquantel in July 2008 while Asamama had no systematic distribution of praziquantel in the years immediately prior to the study. In model 1b, we assessed the agreement between measured hematuria (*Y*_*i*_) and self-reported macrohematuria (*X*_*i*_) collected in Adasawase, Akwaboso, Asamama, Mampong, and Muoso in 2012. In model 1c, we assessed the agreement between measured hematuria (*Y*_*i*_) and self-reported swimming (*X*_*i*_) collected in those same towns in 2012. We used odds ratios with 95% confidence intervals (OR, CI_95%_) to interpret the association between the main predictor and outcome in each model. A supplemental model was also run to assess self-reported macrohematuria and swimming as a vector of predictors (*X*_*i*_). We extracted model-predicted probability values from this supplemental model (i.e., likelihood of correctly predicting infection status via dipstick using self-reported metrics), estimated as the average predicted probability for all groups of children (branches of the classification tree). We used these predicted probabilities to assess whether each self-reported metric contributed to the model's overall ability to predict measured hematuria.

#### 2.6.2. Objective 2: Temporal Stability of Infection Status

In model 2a, we assessed the temporal stability between measured hematuria in 2012 (*Y*_*i*_) and 2010 (*X*_*i*_), collected from Akwaboso, Asamama, Mampong, and Muoso. Data for Adasawase were omitted from the analysis because UGS prevalence was very low in 2010 (<8%), reflecting the presence of an intervention beyond treatment with praziquantel. In model 2b, we assessed the temporal stability between measured hematuria in 2009 (*Y*_*i*_) and 2008 (*X*_*i*_). In model 2c, we assessed the temporal stability between eggs in urine in 2009 (*Y*_*i*_) and 2008 (*X*_*i*_). Data for models 2b and 2c were only available for Adasawase. Similar to Objective 1, statistically significant association between metrics at two time points was assessed using ORs and CI_95%_. We also extracted model-predicted probabilities for models 2a, 2b, and 2c to compare predictive ability of past infection status to current infection status.

## 3. Results

### 3.1. Agreement among Metrics

An exploratory analysis of agreement between* S. haematobium* eggs in urine and measured hematuria showed a statistically significant strong relationship for all groups (chi-square tests: Table S3, Supplemental Material; Kendall's Tau: boys = 0.547, *p* < 0.0001; girls = 0.669, *p* < 0.0001). Regression models, which controlled for age and town, also showed a very stable association between measured hematuria and eggs. For girls, logistic regression analysis showed that the Adj.-OR of having eggs in urine increases if the girl tests positive for measured hematuria (Table 2, model 1a).

An exploratory analysis of agreement between measured hematuria and self-reported metrics demonstrated that self-reported macrohematuria and swimming were significant predictors of measured hematuria for girls (Adj.-OR of 3.2 and 2.4 for self-reported macrohematuria and self-reported swimming, respectively) (Table S5, Supplemental Material). For boys, self-reported macrohematuria was not significantly predictive of measured hematuria, but there appeared to be a weak potential association between self-reported swimming and measured hematuria (Table S5, Supplemental Material). The logistic regression models that followed the exploratory analyses controlled for age and town; they showed that girls who self-report macrohematuria are 4.09 times more likely to have measured hematuria than those who do not self-report macrohematuria (CI_95%_: 2.1–7.9) (Table 2, model 1b). Girls who self-report swimming are 3.6 times more likely to have measured hematuria than nonswimmers (CI_95%_: 1.6–7.9) (Table 2, model 1c). For boys, neither self-reported metric significantly predicted measured hematuria in the logistic regression models (Table 2, models 1b and 1c).

**Figure 3:**
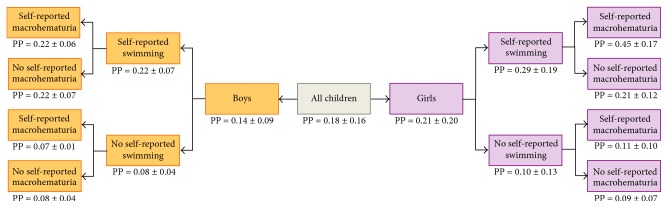
Predicted probability (PP) (mean ± SD) of testing positive for measured hematuria given information about self-reported swimming behavior and self-reported macrohematuria.

Figure 3 shows model-predicted probability (Table S5, Supplemental Material). In this study, a child had an average probability of 0.18 ± 0.16 to test positive for measured hematuria (0.21 ± 0.20 for girls and 0.14 ± 0.09 for boys). For a girl who self-reported swimming only, the probability of a positive dipstick test was 0.21 ± 0.15; the corresponding probability was 0.45 ± 0.22 if she also self-reported macrohematuria. A girl who answered “no” to both questions had a probability of 0.09 ± 0.08 to test positive. Boys who self-reported swimming only or swimming and macrohematuria had about the same probability of testing positive for measured hematuria: 0.22 ± 0.07 and 0.22 ± 0.06, respectively. Boys who self-reported neither macrohematuria nor swimming had a probability of 0.08 ± 0.03 of testing positive.

### 3.2. Infection Status Stability across Time

When controlling for age and town, girls who were positive for measured hematuria in 2010 were 3.3 (CI_95%_: 1.0–10.5) times more likely to be positive in 2012 as well (Table 3, model 2a). A statistically significant association was found between the 2008 and 2009 measured hematuria status for girls (OR = 2.8; CI_95%_: 1.1–6.8) (Table 3, model 2b). However, egg status in 2008 was not predictive of egg status in 2009 in the same data set (Table 3, model 2c). For boys, the presence of either eggs or measured hematuria in 2008 is equally predictive of egg or measured hematuria status in 2009. Controlling for age, boys with eggs in urine in one year are 4.8 times more likely to have eggs in urine in the subsequent year (CI_95%_: 1.2–18.8) and those with measured hematuria in one year are 6.0 times more likely to have measured hematuria in a subsequent year (CI_95%_: 1.5–23.9) (Table 3, models 2b and 2c). A similar association was not observed in the 2010 to 2012 analysis (Table 3, model 2a).

**Figure 4:**
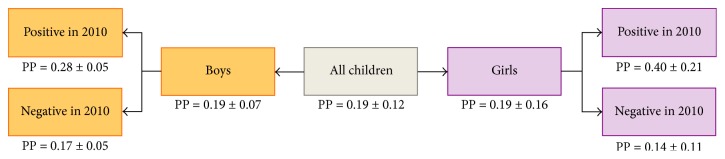
Predicted probability of testing positive for measured hematuria in 2012 as a function of measured hematuria status in 2010 (model 2a).

**Figure 5:**
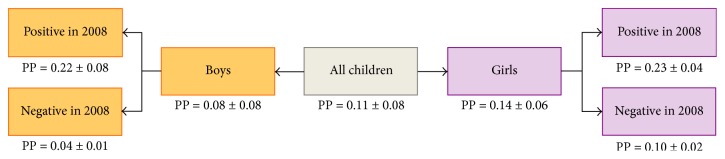
Predicted probability of testing positive for measured hematuria in 2009 as a function of measured hematuria status in 2008 (model 2b).

Knowing an individual's infection status one or two years before offers additional predictive capacity over just knowing their sex (Figures 4 and 5). In analysis 2a (Figure 4), a child had an average predicted probability of 0.19 ± 0.12 to test positive for measured hematuria via dipstick in 2012; this probability was the same for girls and boys (*p* = 0.19 ± 0.16 and 0.19 ± 0.07, resp.). For a girl who was positive by dipstick in 2010, the probability increased to 0.40 ± 0.21, as compared to a girl who was negative in 2010 (*p* = 0.14 ± 0.11). A similar comparison can be made for boys who were positive in 2010 (*p* = 0.28 ± 0.05) versus negative (*p* = 0.17 ± 0.05). Lower overall prevalence in 2009 as compared to 2012 contributed to slightly lower probabilities in Figure 5; the associations between prior (in this case one year before) and current measured hematuria status were maintained for boys and for girls (model 2b). The analogous figure for model 2c is available in the supplemental material (Figure S1, Supplemental Material).

## 4. Discussion

### 4.1. Agreement among Metrics

Very stable associations between measured hematuria and* S. haematobium* eggs for both boys and girls were detected when these metrics were collected at the same point in time (model 1a). Findings differed somewhat for self-reported metrics. Self-reported data collected from girls showed a statistically significant relationship between self-reported macrohematuria and dipstick and between self-reported swimming and dipstick (models 1b and 1c). For boys, neither of the self-reported metrics significantly predicted measured hematuria via dipstick (models 1b and 1c). This suggests that self-reporting may be sufficient for girls when broadly assessing UGS prevalence, but prevalence among boys may need to be checked using parasitological or morbidity measurements. While this is true in the study communities, an assessment of this trend at a broader scale is needed before widely using self-reporting metrics in areas that regularly experience MDA.

Direct comparison of our results with those of others is challenging. In 1995, the Red Urine Study Group published a report discussing the diagnostic accuracy of methods to identify communities in which individuals are likely to experience high morbidity from schistosomiasis. This was a questionnaire-based approach in which teachers asked schoolchildren to self-report symptoms [8]. This approach differs from the work we report here in that the Red Urine group collected data at the level of the classroom and we considered the correlation between metrics at the level of the individual. Moreover, the emphases of the two studies were different, with the Red Urine Study Group seeking to identify high morbidity locations [8] and our study assessing low prevalence communities as well. Self-reported swimming was assessed by Hammad et al. [19] as a risk factor for infection, but the outcome variable was* S. haematobium* ova in urine via filtration, and the precise method of filtration was not described clearly enough to compare with our results; the authors' objective was not to assess self-reported swimming as a proxy for infection status. Satayathum et al. [22] found that there was no association between the frequency of water contact and infection status, but the water contact was observed rather than self-reported, and the variable was the amount of contact (>10 contacts versus ≤10 contacts), not necessarily swimming contact, specifically. Rudge et al. [21] assessed correlation between self-reported swimming/playing and infection status as determined via filtration of 10 mL of urine; they found associations for boys and girls during univariate analyses, but these were not significant in multivariate logistic regression models that included distance to water contact sites from a child's home hamlet; the distance variables were much more predictive of infection status than self-reported swimming/playing. As part of a larger study in Tanzania, Knopp et al. [28] assessed the relationship between* S. haematobium* eggs in 10 mL urine samples and reagent strip readings; they found a significant, strong correlation in children aged 9–12 years, which is the age group most comparable to the age groups we studied. Knopp et al. [28] also found a statistically significant relationship between the color of urine samples and the number of* S. haematobium* eggs in a sample.

In our study, the model used to assess whether the two self-reported metrics (self-reported swimming and macrohematuria) each contributed independent predictive ability (Figure 3, Table S5, Supplemental Material) showed that, for girls, knowing both self-reported metrics provides improvement over knowing just one. For boys, knowing only self-reported swimming status might be sufficient; self-reported macrohematuria status does not change predicted probability of infection. Because of the low cost and ease of collecting this type of data during prevalence studies, there are clear advantages to using self-reported metrics. However, the predictive capacity of these two metrics needs to be evaluated in a larger study with sufficient power to detect the desirable change, which is an important direction for future research. Finally, a limitation to self-reported macrohematuria for girls is that macrohematuria could be confused with menstruation.

The ability to consider the exposure location(s), given the focality of the disease, might provide additional insights into disease transmission. In exploring where the exposure may have occurred, we observed that the communities in our study are very small and all surface water contact locations are within walking distance. Some communities have 9+ water contact sites on a single stream, so it would be challenging to determine which site is which based on a child's verbal description and the data quality could be questionable. Furthermore, in many communities, children swim with friends and might vary their swimming location depending on whom they swim with. Thus, we assumed that by asking questions about swimming/playing in water, we might capture a predictive factor of infection; a future study may explore whether the specific locations and/or the number of river access points have a better predictive capacity.

### 4.2. Temporal Stability of Infection

Model 2a showed that girls who were positive for measured hematuria in 2010 were significantly more likely (Adj.-OR = 3.3, CI_95%_ 1.0–10.5) to be positive again in 2012 than their girl classmates who tested negative for measured hematuria in 2010, although CI_95%_ is somewhat wide. For boys, no significant association was found for measured hematuria between 2010 and 2012. In contrast to model 2a, model 2b was constructed with data collected only one year apart; measured hematuria status in 2008 was predictive of measured hematuria in 2009 for both girls and boys (Adj.-OR = 2.8, CI_95%_ 1.1–6.8; Adj.-OR = 6.0, CI_95%_ 1.5–23.9, respectively), but for boys in particular, CI_95%_ is wide, indicating some uncertainty. The findings from model 2c showed less temporal stability for girls, with no statistically significant association between* S. haematobium* eggs in 2008 and 2009. For boys, there was a significant association (Adj.-OR = 4.8, CI_95%_ 1.2–18.8), but again, a wide confidence interval. In Egypt, Hammad et al. [19] found no association between history of infection and an individual's current infection status, but the authors did not explain how history of infection was determined so results cannot be directly compared with ours. Satayathum et al. [22] found that prior hematuria status measured by dipstick was a significant independent predictor of infection status during a 9-year longitudinal study.

The differences in temporal stability over a period of either one or two years could have various causes. First, they could be due to differences in the study population; the 2010–2012 dataset included low prevalence communities and the 2008-2009 dataset did not. While we constructed the models to account for this factor, it is likely that the small number of towns precluded us from fully controlling for this effect. The differences may also stem from the introduction of national MDA in the study area in recent years. MDA was conducted in 2011, almost exactly a year after the 2010 study and a year before the 2012 study. Finally, there are probably gendered differences in the way children interact with surface water that vary in consistency and intensity over time [22]. Swimming is not the only risk factor for UGS [3, 19]; wading through water and consistent fetching of surface water may be more risky than occasional swimming. Domestic water collection and use are also behaviors that are unlikely to change in the presence of education on how to reduce UGS risk, if accompanying infrastructure is not improved or made more accessible. Future studies should seek to assess gender dynamics and additional aspects of surface water use. Finally, the role played by acquired immunity to schistosomiasis should be further assessed, as this could impact the ability to predict current infections based on previous infections [22]. In a practical sense, and with an eye to Sustainable Development Goal 3.3 [2], our results suggest that broad-scale studies in various geospatial and temporal contexts should be undertaken that follow school-aged children over time in the presence of MDA; it should not be assumed that MDA should be distributed in communities solely because schistosomiasis has historically been endemic or that it is unneeded in communities that have traditionally had low prevalence.

## 5. Conclusions

Our study shows that, in a population of school-aged children where MDA with praziquantel regularly occurs, four different metrics of infection agree for girls and two agree for boys. Thus, where it is affordable and available, the dipstick test can be used for broad-scale prevalence studies with school-aged children, but it is known to underestimate total prevalence unless it is used repeatedly [12], so allowance should be made to offer praziquantel more widely, even to individuals who have not tested positive for measured hematuria. If prevalence data are to be collected via dipstick, we also recommend that data about age, sex, and location be collected; our data shows that performance varies according to these characteristics. The dipstick is a particularly attractive option in comparison with egg filtration, given that cost is generally much lower and it is relatively easy to use. We suggest that self-reported swimming be studied further in broader contexts as a predictor of infection given that it performed reasonably well for girls in our study; if it is found in a larger study to perform well for both boys and girls, this measure could augment self-reported macrohematuria in situations where self-reporting is the only practical option for prevalence assessment. Finally, the observed change in infection status over time is an important tool for disease monitoring; additional large-scale testing should be conducted with both measured hematuria and* S. haematobium* egg data, given that there are differences in how these two metrics perform in boys versus girls and with respect to each other. If these metrics are used to assess the efficacy of disease control strategies, they may be able to provide valuable information about which demographic groups continue to experience transmission over time, so that control measures can be further focused.

## Supplementary Material

Supplementary tables provide additional information about our study. Table S1 shows which metrics were collected during the original primary studies, as well as the praziquantel distribution methods for each study community. Table S2 provides demographic information and information about infection and treatment characteristics of children. Table S3 shows the exploratory analysis of agreement between S. haematobium eggs in urine and hematuria via chi-square tests. Table S4 shows the exploratory analysis of agreement between hematuria and self-reported metrics via chi-square tests. Finally, Table S5 shows the results of a logistic regression model demonstrating the agreement between hematuria and both self-reported metrics, controlling for age and town.

## Figures and Tables

**Table 1:** Descriptive statistics of variables used in each model.

Variables of interest	Boys		Girls		All	
	Total (*N*)	*n* (%)	Total (*N*)	*n* (%)	Total (*N*)	*n* (%)
Model 1a: agreement between measured hematuria and eggs in urine						
Measured hematuria in 2009	308	85 (27.6)	349	105 (30.1)	657	190 (28.9)
Eggs in urine in 2009	308	57 (18.5)	349	86 (24.6)	657	143 (21.8)
Age in years in 2009						
6–10	308	140 (45.5)	349	127 (36.4)	657	267 (40.6)
11–14	308	116 (37.7)	349	148 (42.4)	657	264 (40.2)
15+	308	52 (16.9)	349	74 (21.2)	657	126 (19.2)
Town						
Adasawase	308	185 (60.1)	349	226 (64.8)	657	411 (62.6)
Asamama	308	123 (39.9)	349	123 (35.2)	657	246 (37.4)

Models 1b and 1c: agreement between self-reported macrohematuria and swimming and measured hematuria						
Measured hematuria	292	41 (14.0)	344	72 (20.9)	636	113 (17.8)
Self-reported macrohematuria	292	36 (12.3)	344	77 (22.4)	636	113 (17.8)
Self-reported swimming	292	127 (43.5)	344	198 (57.6)	636	325 (51.1)
Age in years in 2012						
6–10	292	116 (39.7)	344	99 (28.8)	636	215 (33.8)
11–14	292	135 (46.2)	344	162 (47.1)	636	297 (46.7)
15+	292	41 (14.0)	344	83 (24.1)	636	124 (19.5)
Town						
Akwaboso, Mampong, and Muoso	292	121 (41.4)	344	160 (46.5)	636	281 (44.2)
Adasawase	292	84 (28.8)	344	91 (26.5)	636	175 (27.5)
Asamama	292	87 (29.8)	344	93 (27)	636	180 (28.3)

Model 2a: agreement in measured hematuria in 2010 and 2012						
Measured hematuria in 2010	119	25 (21.0)	124	20 (16.1)	243	45 (18.5)
Measured hematuria in 2012	119	23 (19.3)	124	23 (18.5)	243	46 (18.9)
Age in years in 2012						
6–10	119	30 (25.2)	124	22 (17.7)	243	52 (21.4)
11–14	119	62 (52.1)	124	76 (61.3)	243	138 (56.8)
15+	119	27 (22.7)	124	26 (21)	243	53 (21.8)
Town						
Akwaboso, Muoso	119	31 (26.1)	124	39 (31.5)	243	70 (28.8)
Asamama	119	88 (73.9)	124	85 (68.5)	243	173 (71.2)

Models 2b and 2c: agreement between measured hematuria and eggs in urine in 2008 and 2009						
Measured hematuria in 2008	130	27 (20.8)	171	47 (27.5)	301	74 (24.6)
Measured hematuria in 2009	130	10 (7.7)	171	24 (14)	301	34 (11.3)
Eggs in urine in 2008	130	27 (20.8)	171	43 (25.1)	301	70 (23.3)
Eggs in urine in 2009	130	10 (7.7)	171	29 (17)	301	39 (13)
Age in years in 2008						
6–10	130	38 (29.2)	171	42 (24.6)	301	80 (26.6)
11–14	130	69 (53.1)	171	90 (52.6)	301	159 (52.8)
15+	130	23 (17.7)	171	39 (22.8)	301	62 (20.6)

**Table 2:** Results of logistic regression models showing the agreement between prevalence metrics (models 1a, 1b, and 1c).

	Boys		Girls	
	Adj-OR	CI_95%_	Adj-OR	CI_95%_
Model 1a: *Y*_*i*_ = eggs in urine				
Measured hematuria	23.84^d^	**(9.83, 57.80)**	**40.00**	**(18.22, 87.83)**
Ages 11–14^a^	0.94	(0.42, 2.09)	0.90	(0.43, 1.88)
Ages 15+	0.47	(0.18, 1.23)	0.65	(0.26, 1.62)
Town: Asamama^b^	0.89	(0.38, 2.11)	0.79	(0.37, 1.69)

Model 1b: *Y*_*i*_ = measured hematuria				
Self-reported macrohematuria	1.04	(0.39, 2.74)	**4.09**	**(2.12, 7.89)**
Ages 11–14	0.74	(0.35, 1.56)	1.31	(0.67, 2.56)
Ages 15+	1.07	(0.40, 2.88)	0.67	(0.28, 1.62)
Town: Adasawase^c^	1.67	(0.65, 4.31)	**10.68**	**(4.41, 25.91)**
Town: Asamama^c^	**4.30**	**(1.85, 9.99)**	**10.68**	**(4.58, 24.90)**

Model 1c: *Y*_*i*_ = measured hematuria				
Self-reported swimming	2.10	(0.87, 5.05)	**3.60**	**(1.64, 7.94)**
Ages 11–14	0.77	(0.36, 1.63)	0.97	(0.50, 1.88)
Ages 15+	1.21	(0.44, 3.36)	0.63	(0.26, 1.49)
Town: Asamama^c^	1.41	(0.53, 3.75)	**10.91**	**(4.49, 26.49)**
Town: Asamama^c^	2.60	(0.94, 7.02)	**8.14**	**(3.49, 19.02)**

^a^Age group 1 (ages 6–10 years) was the reference category for the “age” variable for all models.

^b^Town group 2 (Adasawase) was the reference category for the “town” variable.

^c^Town group 1 (Akwaboso, Mampong, and Muoso) was the reference category for the “town” variable.

^d^Bold indicates statistical significance at the 0.05 level.

**Table 3:** Results of logistic regression models showing the temporal stability of infection status as assessed by two metrics.

	Boys		Girls	
	OR	CI_95%_	OR	CI_95%_
Model 2a: *Y*_*i*_ = measured hematuria in 2012				
Measured hematuria in 2010	2.0	(0.65, 5.9)	**3.3**	**(1.01, 10.5)**
Age group 2	0.84	(0.27, 2.7)	0.65	(0.18, 2.3)
Age group 3	1.7	(0.45, 6.3)	0.64	(0.13, 3.2)
Town group 3	1.6	(0.47, 5.2)	—^a^	—

Model 2b: *Y*_*i*_ = measured hematuria in 2009				
Measured hematuria in 2008	6.0	**(1.5, 23.9)**	**2.8**	**(1.1, 6.8)**
Age group 2	1.1	(0.18, 6.4)	1.00	(0.35, 2.9)
Age group 3	2.5	(0.38, 16.2)	0.56	(0.14, 2.2)

Model 2c: *Y*_*i*_ = eggs in urine in 2009				
Eggs in urine in 2008	**4.8**	**(1.2, 18.8)**	1.9	(0.79, 4.5)
Age group 2	0.80	(0.17, 3.7)	0.95	(0.37, 2.4)
Age group 3	0.67	(0.09, 4.8)	0.44	(0.12, 1.6)

^a^Standard error is too large for town group 3 in model 2a.

## Additional Points

*Author Summary*. Schistosomiasis affects over 200 million people worldwide. Major symptoms of urogenital schistosomiasis include blood and parasite eggs in urine. Many methods can be used for diagnosis, but diagnosing infection is becoming increasingly difficult due to population-based treatment, which causes a high proportion of “light” infections in which it is more difficult to find eggs or blood. We assessed relationships among parasite eggs, blood in urine, and two self-reported metrics (swimming and visibly bloody urine) and we examined the stability of eggs and blood over time in 5 Ghanaian communities. Using logistic regression models, we found a strong relationship between eggs and bloody urine, which was expected. We also found that self-reported metrics performed relatively well for girls but not boys. The results for longitudinal stability of eggs and blood varied for boys and girls, depending on the metric and the gap between the two assessment times. Given the agreement between the blood and eggs, we recommend the dipstick test for blood for broad use. We recommend that self-reported swimming and self-reported blood in urine be examined further prior to being widely used in the field. Finally, the observed change in infection status over time is an informative tool for disease monitoring and annual dipstick testing should be implemented to monitor and inform disease control strategies.
