# Supplementary material for: Agreement among Four Prevalence Metrics for Urogenital Schistosomiasis in the Eastern Region of Ghana
Source: Biomed Res Int. 2016 Dec 18;2016:7627358. doi: 10.1155/2016/7627358 (PMC5203922; doi:10.1155/2016/7627358)
Supplement: Supplementary file 1 — Supplementary tables provide additional information about our study. Table S1 shows which metrics were collected during the original primary studies, as well as the praziquantel distribution methods for each study community. Table S2 provides demographic information and information about infection and treatment characteristics of children. Table S3 shows the exploratory analysis of agreement between S. haematobium eggs in urine and hematuria via chi-square tests. Table S4 shows the exploratory analysis of agreement between hematuria and self-reported metrics via chi-square tests. Finally, Table S5 shows the results of a logistic regression model demonstrating the agreement between hematuria and both self-reported metrics, controlling for age and town. [file 7627358.f1.docx]

Table S1: Metrics collected during the original primary studies from which data was drawn; praziquantel distribution methods shown for each study community in each year; distribution depended on the prevalence of infection and guidance offered by Ghana Health Service.

|  |  | **Adasawase** | **Asamama** | **Akwaboso** | **Muoso** | **Mampong** |
| --- | --- | --- | --- | --- | --- | --- |
| **2008** | **Metric** | Egg counts |  |  |  |  |
|  |  | Hematuria |  |  |  |  |
|  | **Intervention** | Mass Drug Administration |  |  |  |  |
| **2009** | **Metric** | Eggs | Eggs |  |  |  |
|  |  | Hematuria | Hematuria |  |  |  |
|  | **Intervention** | Mass Drug Administration +WRA | Mass Drug Administration |  |  |  |
| **2010** | **Metric** | Eggs |  |  |  |  |
|  |  | Hematuria | Hematuria | Hematuria | Hematuria |  |
|  | **Intervention** | Positives + Requests | Mass Drug Administration | Positives | Positives |  |
| **2012** | **Metric** | Hematuria | Hematuria | Hematuria | Hematuria | Hematuria |
|  |  | 2 proxies | 2 proxies | 2 proxies | 2 proxies | 2 proxies |
|  | **Intervention** | Positives + Requests | Positives + Requests | Positives | Positives | Positives + Requests |
|  | **Total Years of Data** | 4 | 3 | 2 | 2 | 1 |

Table S2: Demographic, infection, and treatment characteristics of children who were enrolled in school in the various primary studies from which the data was drawn.

| **Town Name** | **Year** | **Enrolled Children** | **Ages Screened** | **Screened** | **Hematuria** | **Total Treated** | **Positives Treated** |
| --- | --- | --- | --- | --- | --- | --- | --- |
|  |  | **Number** | **Range** | **Number (%)** | **Number (%)** | **(%)** | **(%)** |
| **GIRLS** | | | | | | | |
| Adasawase | 2008 | 208 | 8 to 18 | 202 (97.1) | 35 (17.3) | 90.4 | 94.3 |
|  | 2009 | 213 | 6 to 18 | 205 (96.2) | 21 (10.2) | 87.3 | 95.2 |
|  | 2010 | 274 | 3 to 18 | 247 (90.1) | 18 (7.3) | 9.9 | 83.3 |
|  | 2012 | 279 | 3 to 22 | 238 (85.3) | 36 (15.1) | 9.3 | 72.2 |
| Akwaboso | 2010 | 86 | 6 to 17 | 42 (48.8) | 4 (9.5) | 4.7 | 100.0 |
|  | 2012 | 40 | 7 to 14 | 36 (90.0) | 2 (5.6) | 5.0 | 100.0 |
| Asamama | 2009 | 347 | 3 to 18 | 306 (88.2) | 147 (48.0) | 91.1 | 93.9 |
|  | 2010 | 400 | 3 to 19 | 196 (49.0) | 44 (22.4) | 54.0 | 93.2 |
|  | 2012 | 365 | 3 to 19 | 255 (69.6) | 76 (29.8) | 42.2 | 97.4 |
| Mampong | 2012 | 26 | 6 to 16 | 21 (80.8) | 0 (0.0) | 30.8 | n/a |
| Muoso | 2010 | 42 | 7 to 17 | 36 (85.7) | 1 (2.8) | 2.4 | 100.0 |
|  | 2012 | 85 | 7 to 19 | 72 (84.7) | 7 (9.7) | 5.9 | 71.4 |
| **BOYS** | | | | | | | |
| Adasawase | 2008 | 269 | 8 to 19 | 262 (97.4) | 71 (27.1) | 92.2 | 95.8 |
|  | 2009 | 257 | 6 to 21 | 243 (94.6) | 37 (15.2) | 89.1 | 91.9 |
|  | 2010 | 351 | 3 to 22 | 314 (89.5) | 23 (7.3) | 11.4 | 78.3 |
|  | 2012 | 296 | 3 to 21 | 249 (84.1) | 56 (22.5) | 14.5 | 75.0 |
| Akwaboso | 2010 | 111 | 5 to 23 | 63 (56.8) | 6 (9.5) | 9.0 | 100.0 |
|  | 2012 | 64 | 7 to 17 | 54 (84.4) | 1 (1.9) | 1.6 | 100.0 |
| Asamama | 2009 | 415 | 3 to 20 | 363 (87.5) | 183 (50.4) | 87.5 | 90.2 |
|  | 2010 | 506 | 3 to 21 | 239 (47.2) | 41 (17.2) | 52.6 | 90.2 |
|  | 2012 | 428 | 3 to 19 | 264 (61.7) | 89 (33.7) | 41.8 | 97.8 |
| Mampong | 2012 | 48 | 6 to 19 | 37 (77.1) | 4 (10.8) | 37.5 | 75.0 |
| Muoso | 2010 | 58 | 7 to 17 | 48 (82.8) | 4 (8.3) | 5.2 | 75.0 |
|  | 2012 | 95 | 6 to 18 | 80 (84.2) | 3 (3.8) | 3.2 | 100.0 |

Table S3: Exploratory analysis of agreement between *S. haematobium* eggs in urine and hematuria via chi-square tests.

|  |  | **BOYS - *S. haematobium* Eggs** | | | | | | **GIRLS - *S. haematobium* Eggs** | | | | | |
| --- | --- | --- | --- | --- | --- | --- | --- | --- | --- | --- | --- | --- | --- |
|  |  | **no** | **(%)** | **yes** | **(%)** | **χ2 value** | **p-value** | **no** | **(%)** |  | **(%)** | **χ2 value** | **p-value** |
| All | | | | | | | | | | | | | |
| Hematuria | no | 211 | (69) | 12 | (4) | 92.30 | 0.000 | 230 | (66) | 4 | (4) | 156.08 | 0.000 |
|  | yes | 40 | (13) | 45 | (15) |  |  | 33 | (9) | 21 | (21) |  |  |
| Age group 1 | | | | | | | | | | | | | |
| Hematuria | no | 99 | (71) | 7 | (5) | 41.34 | 0.000 | 78 | (61) | 5 | (5) | 54.92 | 0.000 |
|  | yes | 15 | (11) | 19 | (14) |  |  | 13 | (10) | 24 | (24) |  |  |
| Age group 2 | | | | | | | | | | | | | |
| Hematuria | no | 83 | (72) | 5 | (4) | 37.94 | 0.000 | 98 | (66) | 5 | (5) | 61.21 | 0.000 |
|  | yes | 12 | (10) | 16 | (14) |  |  | 14 | (9) | 20 | (20) |  |  |
| Age group 3 | | | | | | | | | | | | | |
| Hematuria | no | 29 | (56) | 0 | (0) | 15.61 | 0.000 | 54 | (73) | 1 | (1) | 40.84 | 0.003 |
|  | yes | 13 | (25) | 10 | (19) |  |  | 6 | (8) | 18 | (18) |  |  |
| Town group 2 | | | | | | | | | | | | | |
| Hematuria | no | 160 | (86) | 7 | (4) | 68.82 | 0.000 | 181 | (80) | 5 | (5) | 99.15 | 0.000 |
|  | yes | 6 | (3) | 12 | (6) |  |  | 9 | (4) | 11 | (11) |  |  |
| Town group 3 | | | | | | | | | | | | | |
| Hematuria | no | 51 | (41) | 5 | (4) | 23.23 | 0.000 | 49 | (40) | 2 | (2) | 45.43 | 0.000 |
|  | yes | 34 | (28) | 33 | (27) |  |  | 24 | (20) | 38 | (38) |  |  |

Table S4: Exploratory analysis of agreement between hematuria and self-reported metrics via chi-square tests.

|  |  | **BOYS - Hematuria via Dipstick** | | | | | | **GIRLS - Hematuria via Dipstick** | | | | | |
| --- | --- | --- | --- | --- | --- | --- | --- | --- | --- | --- | --- | --- | --- |
|  |  | **no** | **(%)** | **yes** | **(%)** | **χ2 value** | **p-value** | **no** | **(%)** | **yes** | **(%)** | **χ2 value** | **p-value** |
| **All** | | | | | | | | | | | | | |
| SR-blood | no | 221 | (76) | 35 | (12) | 0.23 | 0.63 | 227 | (66) | 40 | (12) | 25.51 | 0.00 |
|  | yes | 30 | (10) | 6 | (2) |  |  | 45 | (13) | 32 | (9) |  |  |
| **Age group 1** | | | | | | | | | | | | | |
| SR-blood | no | 81 | (70) | 14 | (12) | 0.00 | 0.96 | 59 | (60) | 10 | (10) | 9.75 | 0.00 |
|  | yes | 18 | (16) | 3 | (3) |  |  | 17 | (17) | 13 | (13) |  |  |
| **Age group 2** | | | | | | | | | | | | | |
| SR-blood | no | 110 | (81) | 14 | (10) | 2.34 | 0.13 | 103 | (64) | 23 | (14) | 8.55 | 0.00 |
|  | yes | 8 | (6) | 3 | (2) |  |  | 21 | (13) | 15 | (9) |  |  |
| **Age group 3** | | | | | | | | | | | | | |
| SR-blood | no | 30 | (73) | 7 | (17) | 0.91 | 0.34 | 65 | (78) | 7 | (8) | 5.89 | 0.02 |
|  | yes | 4 | (10) | 0 | (0) |  |  | 7 | (8) | 4 | (5) |  |  |
| **Town group 1** | | | | | | | | | | | | | |
| SR-blood | no | 100 | (83) | 9 | (7) | 1.07 | 0.30 | 123 | (77) | 7 | (4) | 0.22 | 0.64 |
|  | yes | 12 | (10) | 0 | (0) |  |  | 29 | (18) | 1 | (1) |  |  |
| **Town group 2** | | | | | | | | | | | | | |
| SR-blood | no | 67 | (80) | 8 | (10) | 1.02 | 0.31 | 59 | (65) | 20 | (22) | 5.44 | 0.02 |
|  | yes | 7 | (8) | 2 | (2) |  |  | 5 | (5) | 7 | (8) |  |  |
| **Town group 3** | | | | | | | | | | | | | |
| SR-blood | no | 54 | (62) | 18 | (21) | 0.02 | 0.89 | 45 | (48) | 13 | (14) | 19.41 | 0.00 |
|  | yes | 11 | (13) | 4 | (5) |  |  | 11 | (12) | 24 | (26) |  |  |
| **All** | | | | | | | | | | | | | |
| SR-swim | no | 152 | (52) | 13 | (4) | 11.94 | 0.00 | 132 | (38) | 14 | (4) | 19.71 | 0.00 |
|  | yes | 99 | (34) | 28 | (10) |  |  | 140 | (41) | 58 | (17) |  |  |
| **Age group 1** | | | | | | | | | | | | | |
| SR-swim | no | 59 | (51) | 4 | (3) | 7.61 | 0.01 | 41 | (41) | 4 | (4) | 9.52 | 0.00 |
|  | yes | 40 | (34) | 13 | (11) |  |  | 35 | (35) | 19 | (19) |  |  |
| **Age group 2** | | | | | | | | | | | | | |
| SR-swim | no | 69 | (51) | 5 | (4) | 5.07 | 0.02 | 48 | (30) | 4 | (2) | 10.60 | 0.00 |
|  | yes | 49 | (36) | 12 | (9) |  |  | 76 | (47) | 34 | (21) |  |  |
| **Age group 3** | | | | | | | | | | | | | |
| SR-swim | no | 24 | (59) | 4 | (10) | 0.48 | 0.49 | 43 | (52) | 6 | (7) | 0.11 | 0.75 |
|  | yes | 10 | (24) | 3 | (7) |  |  | 29 | (35) | 5 | (6) |  |  |
| **Town group 1** | | | | | | | | | | | | | |
| SR-swim | no | 92 | (76) | 8 | (7) | 0.26 | 0.61 | 80 | (50) | 1 | (1) | 4.90 | 0.03 |
|  | yes | 20 | (17) | 1 | (1) |  |  | 72 | (45) | 7 | (4) |  |  |
| **Town group 2** | | | | | | | | | | | | | |
| SR-swim | no | 49 | (58) | 4 | (5) | 2.60 | 0.11 | 48 | (53) | 10 | (11) | 11.84 | 0.00 |
|  | yes | 25 | (30) | 6 | (7) |  |  | 16 | (18) | 17 | (19) |  |  |
| **Town group 3** | | | | | | | | | | | | | |
| SR-swim | no | 11 | (13) | 1 | (1) | 2.12 | 0.15 | 4 | (4) | 3 | (3) | 0.03 | 0.86 |
|  | yes | 54 | (62) | 21 | (24) |  |  | 52 | (56) | 34 | (37) |  |  |

Table S5: Results of a logistic regression model showing the agreement between hematuria and both self-reported metrics, controlling for age and town.

|  | **BOYS** | | **GIRLS** | |
| --- | --- | --- | --- | --- |
| **Hematuria via dipstick** | **Adj-OR** | **95% CI** | **Adj-OR** | **95% CI** |
| Self-reported hematuria | 0.93 | (0.35, 2.50) | **3.23** | **(1.63, 6.41)** |
| Self-reported swimming | 2.12 | (0.88, 5.12) | **2.44** | **(1.06, 5.63)** |
| Age group 2 | 0.77 | (0.36, 1.62) | 1.11 | (0.55, 2.21) |
| Age group 3 | 1.21 | (0.44, 3.35) | 0.69 | (0.29, 1.67) |
| Town group 2 | 1.41 | (0.53, 3.75) | **12.04** | **(4.85, 29.89)** |
| Town group 3 | 2.60 | (0.94, 7.20) | **8.24** | **(3.46, 19.64)** |
